# Supplementary material for: Hashimoto’s Thyroiditis Does Not Influence the Malignancy Risk in Nodules of Category III in the Bethesda System
Source: Cancers (Basel). 2022 Apr 13;14(8):1971. doi: 10.3390/cancers14081971 (PMC9025593; doi:10.3390/cancers14081971)
Supplement: Supplementary file 1 [file cancers-14-01971-s001.zip › cancers-1651050-supplementary.pdf]

Supplementary Materials: Hashimoto's Thyroiditis Does Not Influence the Malignancy Risk in Nodules of Category III in the Bethesda System

Dorota Słowińska-Klencka, Bożena Popowicz and Mariusz Klencki

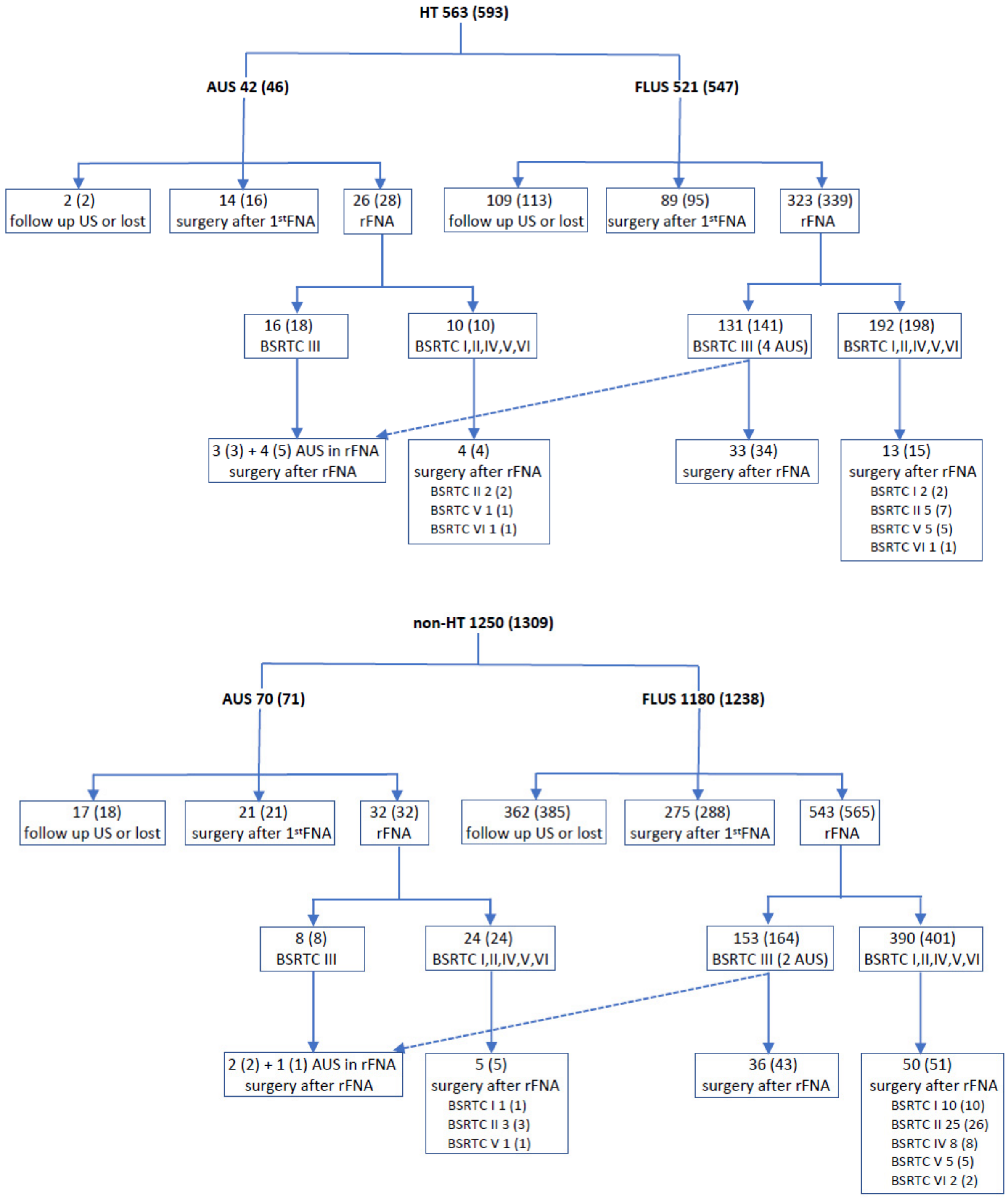

Figure S1. Distribution of surgically treated patients and their nodules between examined groups regarding their rFNA (repeat FNA) status and the initial diagnosis (FLUS vs. AUS). Numbers of analyzed nodules were given in parentheses.

Table S1. Results of repeat FNA of nodules in HT and non-HT groups.

category of repeat FNA	No/% of nodules with repeat FNA		
	**HT 367**	**non-HT 597**	p
I	24/6.5	79/13.2	0.0011
II	176/48.0	329/55.1	0.0309
III	159/43.3	172/28.8	<0.0001
IV	0/0.0	8/1.3	0.0627
V	6/1.6	7/1.2	0.5456
VI	2/0.5	2/0.3	0.9812

Table S2. Results of repeat FNA of nodules in HT and non-HT groups for AUS and FLUS nodules separately

	No/% of nodules with repeat FNA					
**category of repeat FNA**	**HT**			**non-HT**		
	**AUS 28**	**FLUS 339**	**p**	**AUS 32**	**FLUS 565**	**p**
I	1/3.6	23/6.8	0.7923	4/12.5	75/13.3	0.8868
II	7/25.0	169/49.9	0.0114	19/59.4	310/54.9	0.6180
III	18/64.3	141/41.6	0.0199	8/25.0	164/29.0	0.6246
AUS	14/50.0	6/1.8	<0.0001	4/12.5	2/0.3	<0.0001
FLUS	4/14.3	135/39.8	0.0074	4/12.5	162/28.7	<0.0745
IV	-	-		-	8/1.4	0.9104
V	1/3.6	5/1.5	0.9478	1/3.1	6/1.1	0.8322
VI	1/3.6	1/0.3	0.3535	-	2/0.3	0.2167

Table S3. Risk of malignancy (RoM) of nodules in HT and non-HT groups with AUS and FLUS nodules analyzed in three sets of nodules: (A) the whole examined cohort, (B) only nodules without repeat FNA, and (C) only nodules with category III in repeat FNA. RoM was evaluated as a range with its lower limit defined by the number of cancers to the total number of nodules (A1, B1 and C1) and its upper limit defined by the number of cancers to the number of operated nodules (A2, B2 and C2).

Nodules were regarded as an AUS nodule when AUS was diagnosed in at least one FNA (first or repeat one)

Set of nodules and RoM range limit	Risk of malignancy (RoM) [%] (number of nodules with cancer)											
	**All**			**HT**			**non-HT**			**All**		
	**HT**	**non-HT**	**p**	**AUS**	**FLUS**	**p**	**AUS**	**FLUS**	**p**	**AUS**	**FLUS**	**p**
**A1 – lower limit** **all nodules** HT: 593, non-HT: 1309	5.1 (30)	4.5 (59)	0.5976	23.5 (12)	3.3 (18)	<0.0001	16.4 (12)	3.8 (47)	<0.0001	19.4 (24)	3.7 (65)	<0.0001
**A2 – upper limit** **operated nodules** HT: 172, non-HT: 411	17.4 (30)	14.4 (59)	0.3447	42.9 (12)	12.5 (18)	0.0001	41.4 (12)	12.3 (47)	<0.0001	42.1 (24)	12.4 (65)	<0.0001
**B1– lower limit** **all nodules without repeat FNA** HT: 226, non-HT: 712	8.0 (18)	5.5 (39)	0.1727	33.3 (6)	5.8 (12)	<0.0001	17.9 (7)	4.8 (32)	0.0004	22.8 (13)	5.0 (44)	<0.0001
**B2 – upper limit** **nodules operated without repeat FNA** HT: 111, non-HT: 309	16.2 (18)	12.6 (39)	0.3429	37.5 (6)	12.6 (12)	0.0125	33.3 (7)	11.1 (32)	0.0031	35.1 (13)	11.5 (44)	0.0001
**C1– lower limit** **all nodules with category III in repeat FNA** HT: 159, non-HT: 172	4.4 (7)	4.7 (8)	0.9135	17.4 (4)	2.2 (3)	0.0063	20.0 (2)	3.7 (6)	0.1093	18.2 (6)	3.0 (9)	0.0001
**C2 – upper limit** **nodules operated after category III in repeat FNA** HT: 42, non-HT: 46	16.7 (7)	17.4 (8)	0.9281	50.0 (4)	8.8 (3)	0.0223	66.7 (2)	14.0 (6)	0.1233	54.5 (6)	11.7 (9)	0.0004

Table S4. Comparison of the incidence of particular types of cancer HT and non-HT groups.

Type of malignancy [number/%]	all 87		HT 28		non-HT 59	
	HT 28	non-HT 59	AUS 10	FLUS 18	AUS 12	FLUS 47
PTC	19/67.9	33/55.9	7/70.0	12/66.7	10/83.3	23/48.9
NIFTP	1/3.6	-	-	1/5.6	-	-
FTC	3/10.7	8/13.6	1/10.0	2/11.1	-	8/17.0
FT-UMP	1/3.6	3/5.1	-	1/5.6	-	3/6.4
HTC	-	7/11.9	-	-	-	7/14.9
PDTC	-	1/1.7	-	-	-	1/2.1
ATC	1/3.6	2/3.4	-	1/5.6	1/8.3	1/2.1
MTC	2/7.1	2/3.4	1/10.0	1/5.6	-	2/4.3
ML	1/1.7		1/10.0	-	-	-
ANG	-	1/1.7	-	-	1/8.3	-
PC	-	1/1.7	-	-	-	1/2.1
ST	--	1/1.7	-	-	-	1/2.1

PTC—papillary thyroid carcinoma, NIFTP - non-invasive follicular thyroid neoplasm with papillary like nuclear features, FTC follicular thyroid carcinoma, FT-UMP—follicular tumor of uncertain malignant potential, HTC—Hurthle cell thyroid carcinoma, PDTC—poorly differentiated thyroid carcinoma, ATC—anaplastic thyroid carcinoma, MTC—medullary thyroid carcinoma, ML – malignant lymphoma, ANG—angiosarcoma, PC—parathyroid carcinoma, ST—secondary tumor
